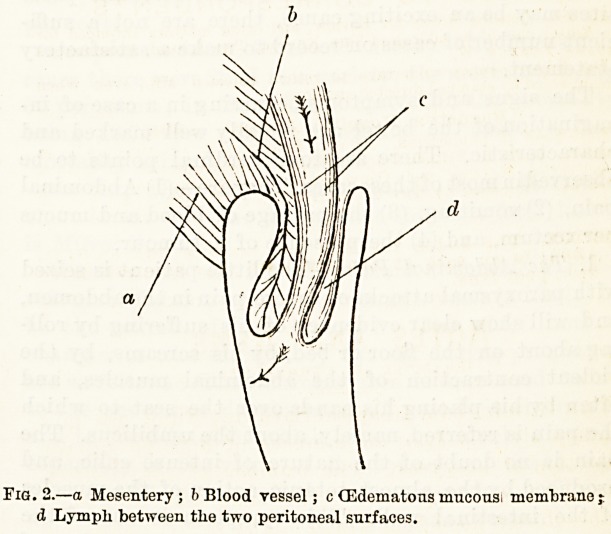# Acute Intussusception. I

**Published:** 1894-06-09

**Authors:** W. McAdam Eccles

**Affiliations:** Assistant Surgeon to the West London Hospital, Assistant Demonstrator of Anatomy at St. Bartholomew's Hospital, Assistant Surgeon to the City of London Truss Society, and Surgeon to the St. Marylebone General Dispensary


### Medical Progress and Hospital Clinics.

[*The Editor will be glad to receive offers of co-operation and contributions from members of the profession. All letters should be addressed to* The Editor, The Lodge, Porchester Square, London, W.]

### ACUTE INTUSSUSCEPTION.—I.

By W. McAdam Eccles, M.B., B.S.Lond., F.R.C.S., Eng., Assistant Surgeon to the West London Hospital, Assistant Demonstrator of Anatomy at St. Bartholomew's Hospital, Assistant Surgeon to the City of London Truss Society, and Surgeon to the St. Marylebone General Dispensary.

Acute intussusception is the invagination of a portion of bowel into the lumen of the intestine immediately below it, with consequent intestinal obstruction of an acute character. I purpose in two papers to deal with the more important and practical features in the symptoms, diagnosis, and treatment of this disease, which is comparatively frequent in young children, and which forms a very large percentage of deaths due to obstruction of the bowel.

Chronic intussusception is not nearly so common as the acute form, and occurs more usually in adults than in children.

Given a child under two years of age with the symptoms of acute intestinal obstruction, in which no evidence of strangulated hernia can be found, its condition is almost certainly due to either invagination of intestine, or its strangulation by a band within the abdomen, very commonly a persistent Meckel's diverticulum. The situation of all others where intussusception is most prone to occur is at the junction of the small with the large intestine, in other words at the ileo-cæcal valve, which, in the commonest variety the ileo-cæcal, forms the apex of the invaginated portion, called the intussusceptum. (Fig. 1.)

Fig. 1.—*a* Entering layer; *b* Returning layer; *c* Sheath. The Entering and Returning Layers from the Intussusceptum.

The causes of the greater frequency here are probably the anatomical condition, that of a smaller portion joining a considerably larger part, and the fact that the valve may act as a partial barrier to the onward passage of intestinal contents, especially if solid, or semi-solid, and so produce a tendency to invagination.

Reference to Figs. 1 and 2 will refresh the memory as to the disposition of the different parts concerned in the condition, and no further reference to this part of the subject in detail will be necessary.

When one length of gut slips into, or more correctly speaking, is forced into another, two results will naturally follow: (1) Obstruction to the lumen of the intestine, with the consequent train of symptoms due to the arrested contents. This is brought about in

Fig. 2.—*a* Mesentery; *b* Blood vessel; *c* Œdematous mucous membrane; *d* Lymph between the two peritoneal surfaces.

three ways, chiefly by the intussusceptum itself acting as a plug within the gut, but also by the swelling of the intestinal walls which occurs, and the usual turning of the apex of the included bowel against the side wall of the sheath, owing to the dragging of the mesentery. (Fig. 2.) All that the increased energetic peristalsis set up by the obstruction does is to increase the amount of the invaginated intestine. (2) Strangulation, that is interference with the circulation through the vessels of the portion of intestine which is within the including part.

The drawing in of the mesentery causes it to become kinked and its vessels consequently pressed upon, with the result that there is great difficulty in the return of the blood through the veins, or even a stoppage of the entrance of the arterial stream. The former condition will lead to intense congestion and swelling, and if persistent to gangrene, the latter to anæmia, and probably also to gangrene of the intussusceptum. In connection with the further pathological conditions which occur, much stress is commonly laid in the text books on the fact that very quickly there is plastic lymph thrown out from the peritoneal surfaces which are in contact (Fig 2), leading to the glueing together of the returning layer and the sheath. That this does happen is no doubt true in many cases, but owing to the tendency for a continuous increase in the amount of bowel invaginated there is not sufficient undisturbed apposition of the part to allow the adhesion to become very strong, so that the gut at any rate in a large number of cases of less than forty-eight hours' duration can be fairly easily reduced.

The causes of intussusception are probably numerous. Irregular peristaltic action is possibly very potent in its production, but there is usually some exciting factor to create the abnormal contraction of the intestinal wall. Some slight obstruction, as mentioned above, occurring at the situation of the ileocæcal valve may have considerable influence in causing the small intestine to pass into the large. A polypus growing from the wall of the gut also may act as a stimulus. It is interesting that most cases do not, as is often asserted, present previous evidence of gastro-enteritis, and therefore may usually be easily diagnosed from that condition. As to whether intestinal parasites may be an exciting cause, there are not a sufficient number of cases on record to make a satisfactory statement.

The signs and symptoms occurring in a case of invagination of the bowel are usually well marked and characteristic. There are four cardinal points to be observed in most of these cases. They are—(1) Abdominal pain, (2) vomiting, (3) the passage of blood and mucus per rectum, and (4) the presence of a tumour.

1. *The Abdominal Pain.*—The little patient is seized with paroxysmal attacks of severe pain in the abdomen, and will show clear evidences of his suffering by rolling about on the floor or bed, by his screams, by the violent contraction of the abdominal muscles, and often by his placing his hands over the seat to which the pain is referred, namely, about the umbilicus. The pain is no doubt of the nature of intense colic, and produced by the almost tetanic action of the muscles of the intestinal wall. This symptom is one of the very earliest, and often leads to the administration of purgatives without the advice of a medical practitioner, such, however, only tending to increase the trouble instead of relieving it. The pain is sooner or later accompanied in most cases by

2. *Vomiting,* but this symptom is sometimes very late in appearing. Owing probably to the obstruction being commonly at the junction of the small with the large intestine, and therefore comparatively low down in the bowel. It is prone to come on after the child has been fed, and the vomit consists first of the contents of the stomach, then of the upper part of the small intestine, being bilious in character, and, lastly, it becomes stercoraceous, sometimes containing blood towards the end. This constant vomiting has a very depressing effect on young children, and is often a prominent factor in the production of the collapse which speedily sets in, and carries off the patient.

3. *The passage of blood and mucus per rectum* is a symptom of pre-eminent importance. Owing to the intense congestion of the invaginated portion of gut alluded to above, blood is exuded into the cavity of the bowel, and from the great irritation produced by the abnormal condition of things mucus in large quantity is secreted, this together with the blood being passed from the bowel, accompanied at first by some fæcal matter, later by flatus only, and finally when obstruction is practically complete the blood-stained mucus is all that comes through the anus. About 75 per cent. of cases have such discharge. If the intussusception has advanced far into the large intestine some tenesmus may attend this symptom.

4. *The Presence of a Tumour.*—Two-thirds of all acute cases present this characteristic sign of a tumour in the abdomen associated with the foregoing symptoms. As invagination usually commences in the region of the ileo-cæcal valve, the tumour can generally be felt in an early stage of the disease in the right iliac fossa, which should, therefore, be always carefully examined. It will then in most cases travel with the increase of the intussuscepted portion of bowel across the abdomen obliquely, and reach the left upper part. From here it descends to the left iliac fossa, and the valve at the apex of the intussusceptum may even protrude from the anus. Unless care be taken in examining the abdomen for this tumour, it may be overlooked, owing chiefly to two causes, firstly the rigid contraction of the muscles of the abdominal wall, and, secondly, the distended condition of the abdomen. An anæsthetic is often advisable during the manipulation. An exploration of the rectum by the finger should never be omitted. The tumour is produced partly by the swollen condition of the affected portion of the gut and partly by the thickness caused by the slipping of one piece into another. The diagnosis, prognosis, and treatment of this interesting disease must be made the substance of a second article.

## Figures and Tables

**Fig. 1. f1:**
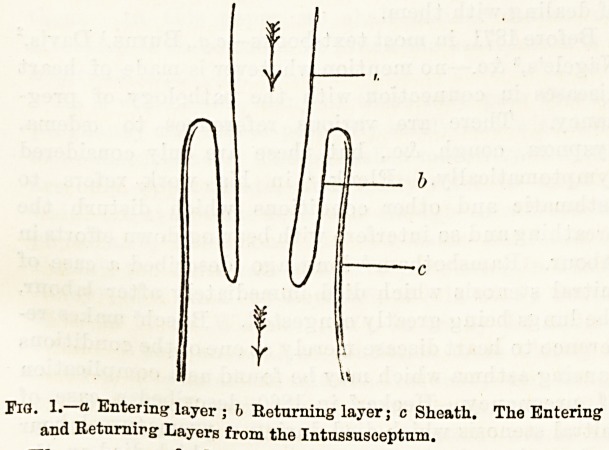


**Fig. 2. f2:**